# Insights into the Two Most Common Cancers of Primitive Gut-Derived Structures and Their Microbial Connections

**DOI:** 10.3390/medicina60091515

**Published:** 2024-09-18

**Authors:** Amitabha Ray, Thomas F. Moore, Dayalu S. L. Naik, Daniel M. Borsch

**Affiliations:** 1 School of Health Professions, D’Youville University, 320 Porter Ave, Buffalo, NY 14201, USA; 2 College of Health Sciences, Glenville State University, Glenville, WV 26351, USA; thomas.moore@glenville.edu; 3 ICMR National Institute of Traditional Medicine, Belagavi 590010, India; daylu.sl@icmr.gov.in; 4 Lake Erie College of Osteopathic Medicine at Seton Hill, Greensburg, PA 15601, USA; dborsch@lecom.edu

**Keywords:** colorectal cancer, pulmonary neoplasm, *Chlamydia* species, *Escherichia coli*, phage therapy

## Abstract

The gastrointestinal and respiratory systems are closely linked in different ways, including from the embryological, anatomical, cellular, and physiological angles. The highest number (and various types) of microorganisms live in the large intestine/colon, and constitute the normal microbiota in healthy people. Adverse alterations of the microbiota or dysbiosis can lead to chronic inflammation. If this detrimental condition persists, a sequence of pathological events can occur, such as inflammatory bowel disease, dysplasia or premalignant changes, and finally, cancer. One of the most commonly identified bacteria in both inflammatory bowel disease and colon cancer is *Escherichia coli*. On the other hand, patients with inflammatory bowel disease are at risk of several other diseases—both intestinal (such as malnutrition and intestinal obstruction, besides cancer) and extraintestinal (such as arthritis, bronchiectasis, and cancer risk). Cancers of the lung and colon are the two most common malignancies occurring worldwide (except for female breast cancer). Like the bacterial role in colon cancer, many studies have shown a link between chronic *Chlamydia pneumoniae* infection and lung cancer. However, in colon cancer, genotoxic colibactin-producing *E. coli* belonging to the B2 phylogroup may promote tumorigenesis. Furthermore, *E. coli* is believed to play an important role in the dissemination of cancer cells from the primary colonic site. Currently, seven enteric pathogenic *E. coli* subtypes have been described. Conversely, three *Chlamydiae* can cause infections in humans (*C. trachomatis* may increase the risk of cervical and ovarian cancers). Nonetheless, striking genomic plasticity and genetic modifications allow *E. coli* to constantly adjust to the surrounding environment. Consequently, *E. coli* becomes resistant to antibiotics and difficult to manage. To solve this problem, scientists are thinking of utilizing suitable lytic bacteriophages (viruses that infect and kill bacteria). Several bacteriophages of *E. coli* and *Chlamydia* species are being evaluated for this purpose.

## 1. Introduction

The gastrointestinal and respiratory systems are closely connected anatomically and physiologically. Our respiratory system develops about the third week of embryonic life, when an outgrowth appears from the ventral wall of the primitive foregut. The endodermal cells of the foregut invade the surrounding mesenchyme and sequentially form the trachea, bronchial tree, and lobules [1]. Therefore, one can observe many similarities and cooperation between the respiratory and gastrointestinal systems. Apart from the pharynx, which belongs to both systems, other essential functions include the maintenance of cellular metabolism and survival by the constant supply of oxygen and nutrients, participation in the elimination of waste products such as carbon dioxide and undigested food materials, and support in the immune response. Of note, the gut-associated lymphatic tissue (GALT), including Peyer’s patches of the ileum, as well as the bronchus-associated lymphatic tissue (BALT) and other lymphatic tissue of the respiratory system, are located in the lamina propria and submucosa, in both diffuse and nodular forms. Histologically, a sizable portion of both gastrointestinal and respiratory tracts are lined by columnar cells and mucous-secreting cells/Goblet cells. Interestingly, a small proportion of the cell population (less than 1%) are neuroendocrine cells, such as small granule cells (Kulchitsky cells) of the respiratory system and enteroendocrine cells of the gastrointestinal system, which are present individually all over the gastrointestinal epithelium and in accumulations in the pancreatic islets of Langerhans [2]. Neoplastic transformation of these cells could release hormonal substances and result in distinct clinical syndromes.

The majority of neuroendocrine neoplasms originate from the gastrointestinal (~70%) and respiratory (~20%) systems; hence, these neoplasms from the two sites account for roughly 90% of all neuroendocrine neoplasms, which overall represent about 0.5% of all malignancies [3,4]. However, aggressive malignancies are more common in the respiratory system [4]. Among pulmonary neuroendocrine neoplasms, small-cell lung carcinoma accounts for approximately 15% of lung primary cancers [5]. Like the association between cigarette smoking and small-cell lung carcinoma, recent reports have shown a connection between gastrointestinal neuroendocrine neoplasms and inflammatory bowel disease [6,7,8,9].

Inflammatory bowel disease, which primarily refers to ulcerative colitis and Crohn’s disease, is a chronic inflammatory disorder of idiopathic origin. Many investigators have reported that patients with inflammatory bowel disease are at risk of the development of malignancies in several sites—both intestinal and extraintestinal, e.g., the colon (colorectal), oral cavity, breast, uterine cervix, skin, and lung [10,11,12,13]. It is worth mentioning that in inflammatory bowel disease, other non-cancerous extraintestinal manifestations may include respiratory tract involvement, such as bronchiectasis, chronic bronchitis, and interstitial pneumonia [14]. Nevertheless, it is believed that inflammatory bowel disease has a link to a number of pathological factors—for instance, genetic susceptibility, environmental elements, abnormal immune response, and alterations in intestinal microbiota (dysbiosis). From this perspective, researchers have categorized several suspected bacterial species, including *Clostridium difficile*, *Mycobacterium avium paratuberculosis*, *Escherichia coli*, *Klebsiella pneumoniae*, *Campylobacter* spp., and *Chlamydia* spp. [15,16].

The prevalence of inflammatory bowel disease is increasing worldwide, particularly in newly industrialized nations [10]. Similarly, an increasing incidence rate is observed globally for another condition, i.e., proctitis/proctocolitis due to lymphogranuloma venereum, which may mimic inflammatory bowel disease [17,18,19]. Lymphogranuloma venereum, caused by *Chlamydia trachomatis*, is a sexually transmitted disease, and relevant proctocolitis is diagnosed specifically in homosexual patients. In a study in Switzerland, the investigators analyzed inflamed biopsy specimens from patients with Crohn’s disease (*n* = 39) and ulcerative colitis (*n* = 13) [20]. In Crohn’s disease-inflamed tissue specimens, significantly more *Chlamydia pneumoniae* DNA was detected compared with specimens from unaffected areas. A study from New Zealand identified *C. pneumoniae* DNA from 21.4% of biopsy specimens from Crohn’s disease (9/42), 15.3% of biopsies from ulcerative colitis (9/59), and 11.4% of biopsies from subjects without inflammatory bowel disease (control, 14/122) [21]. Interestingly, *Chlamydiae* in humans and many animals colonize the gastrointestinal tract, which could be a reservoir for reinfection [22,23].

As mentioned earlier, inflammatory bowel disease is linked to several pathophysiological events, e.g., dietary factors, the compromise of gut mucus tissue integrity, and host immune responses, as well as alterations in microbial diversity and their metabolites [24,25] (Table 1 [26,27,28,29,30,31,32,33]). Among the pathogenic bacteria in this dysbiosis–inflammation–dysplasia–carcinogenesis process, *E. coli* perhaps plays an important role [24,34]. A recent report, which analyzed *E. coli* genomes from patients with Crohn’s disease, ulcerative colitis, a pouch (caused by ileoanal anastomosis in ulcerative colitis), and healthy persons, observed that no strains were unique to inflammatory bowel disease, while *E. coli* B2 phylogenetic group/lineage was more prevalent in ulcerative colitis than in other subjects [35]. Furthermore, *E. coli* strains isolated from ulcerative colitis encoded more genotoxic colibactin, which could increase cancer risk.

**Figure 1:**
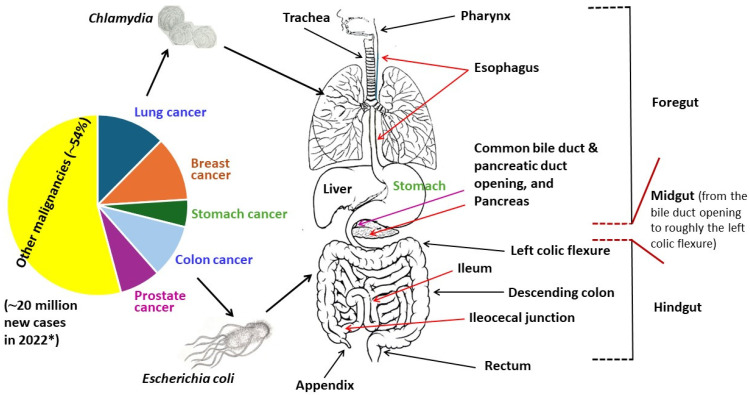
An overview of the global cancer burden and relevant adult derivatives of the primitive gut tube. * Source: The World Health Organization (News release: 1 February 2024). The midgut-hindgut junction is situated between the right two-thirds and the left third of the transverse colon (which extends from the hepatic or right colic flexure to the left colic flexure).

In this review, an attempt has been made to discuss the pathological impacts of *E. coli* and *Chlamydia* in two major neoplastic diseases—colon cancer and lung cancer, respectively (Figure 1). As stated before, tissues of these two sites share the same embryonic origin. Of note, the colon originates from the middle (midgut) and caudal (hindgut) segments of the primitive gut. In addition, relevant antimicrobial resistance and the prospect of phage therapy will be addressed briefly. Unlike *E. coli*, which is a common Gram-negative bacillus and can survive in an open environment, *Chlamydia* is deficient in several biosynthetic/metabolic components, which must be acquired from the host cell.

In this review, an electronic literature search was carried out primarily using PubMed, and, initially, papers published between 2000 and the current year (i.e., 2024) were considered. However, during our comprehensive search and examination of cross-references, we found a few interesting articles that were either published before 2000 or not in English. We utilized the Google system to translate those articles into English. In this process, we also included information from two books and an article that was identified through a Google search. Two authors independently screened the articles/studies, assessed their quality, and extracted the necessary information. The majority of the articles cited in this review were published within the last five years, constituting roughly 59% of the references. This review comprises four main sections: Introduction, *E. coli*-related pathologies, *Chlamydial* infections, and Bacteriophage aspects. We selected relevant articles for each section to ensure a coherent and rational discussion. Overall, our study on both lung and colon cancers has found that microbiota communities play a significant role in human health, including homeostasis and immune function.

## 2. The Large Intestine: *E. coli* and Cancer

**Figure 2:**
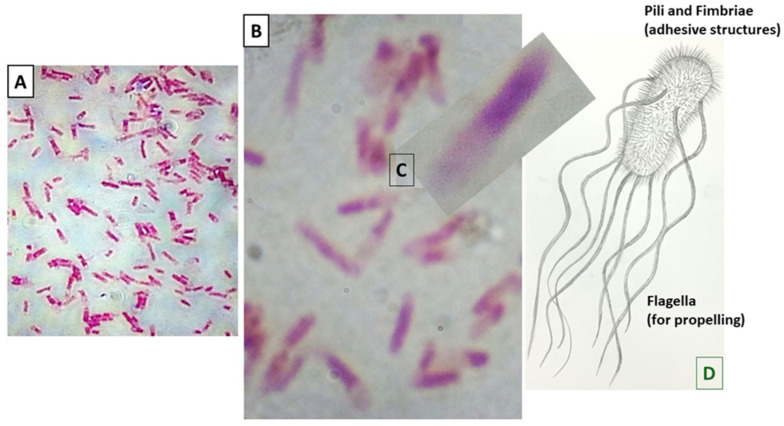
Gram-negative *Escherichia coli* and different surface structures. (**A**) Microscopic view of *E. coli* (100×, oil-immersion). (**B**) Magnification of a part of the microscopic view of (**A**) by the camera system (10 times). (**C**) Enlargement of an *E. coli* to show bacterial appendages (manual expansion and placed obliquely, according to the position of hand-drawn *E. coli* in (**D**)). (**D**) Sketch of an *E. coli*.

*E. coli* is a highly diverse bacterial species—from a commensal organism (without causing any harm to its hosts) to a pathogen for a range of diseases, e.g., infections of the gastrointestinal tract, urinary tract, central nervous system, and bloodstream (Figure 2). Of note, *E. coli* is the most common cause of urinary tract infections. The organism has striking genomic plasticity, which is responsible for its large variability [36]. Genetic modifications such as horizontal gene transfer, point mutations, and DNA rearrangements allow the bacterium to continually adapt to the surrounding environment. Interestingly, within the classical *E. coli*, hybrid- and hetero-pathogenic *E. coli* have been described as indicating a unique arrangement of virulence factors; for instance, Shiga toxin-producing *E. coli* (STEC, which was traditionally not documented) [37]. It may be worth mentioning that multidrug-resistant *E. coli* strains are prevalent in different parts of the world. For example, in Asia, CTX-M-producing and New Delhi metallo-β-lactamase (NDM)-producing *E. coli* strains become a serious concern [38]. Notably, CTX-M is an enzyme under the extended-spectrum β-lactamases (ESBLs), which can hydrolyze β-lactam antibiotics such as cephalosporins and monobactams. On the other hand, many investigators believe that *E. coli* could play a significant role in colon cancer [39,40,41]. Several bacterial characteristics, e.g., induction of chronic inflammation, intracellular parasitism, and production of colibactin, as well as the ability to cause DNA damage and accumulate mutations in host cells, may promote cancer development [34,41,42].

It is commonly mentioned that colibactin-producing *E. coli* can incite carcinogenesis in the colon. Colibactin can cause DNA damage, and this genotoxic metabolite is encoded by 19 genes in a 54 kb polyketide synthase (*pks*) pathogenicity island frequently harbored by *E. coli* from the B2 phylogroup. A Japanese study on 413 patients with colon cancer showed that *pks*+ *E. coli* was more pronounced in tumor tissue from early disease stages [43]. The investigators concluded that *pks*+ *E. coli* may participate in the initial tumor development, but not in tumor progression. In the same manner, the results of the study conducted by Chen et al. have supported the role of *pks*+ *E. coli* in early tumorigenesis [44]. Furthermore, they noticed that their findings might denote additional contributing factors for colon carcinogenesis. It is notable that the development of colon cancer is influenced by a number of risk factors, including some modifiable/lifestyle-related factors, for example, consumption of red meats and processed meats, saturated fat, and alcohol, smoking, insulin resistance/hyperinsulinemia, obesity, and medical interventions such as cholecystectomy [45,46]. A recent study in China documented the fact that *pks*+ *E. coli* was enhanced in patients with cholelithiasis or cholecystectomy [47]. Another study in France showed that colibactin-producing *E. coli* was linked to alterations in the lipid metabolism of cancer cells, which could create an immune-suppressive tumor microenvironment and disease recurrence [48]. The accumulation of lipids in the tumor region might change the intracellular signaling systems, leading to cell proliferation and resistance to chemotherapeutic agents. Interestingly, colibactin-producing *E. coli* has been reported to display resistance to different antibiotics and induce the emergence of tumor cells that exhibit resistance to chemotherapeutic drugs [49,50].

A study that analyzed mucosal *E. coli* isolates from 61 colon cancer patients, along with 20 healthy controls, noticed a trend of a higher rate of colibactin-producing *E. coli* among cancer patients in comparison with controls, but the significance was borderline [51]. However, the investigators of this study observed a higher prevalence of other genes that encode virulence factors such as S-fimbriae, siderophore receptor, invasin, and uropathogenic-specific protein/genotoxin in *E. coli* from cancer patients’ mucosal biopsies. It may be noteworthy that phylogroup B2 *E. coli* also releases other virulence factors collectively called cyclomodulins, which include cytotoxic necrotizing factor, cytolethal distending toxin, and cycle inhibiting factor (apart from colibactin), as well as cyclooxygenase–2 [52,53]. On the other hand, in an in vitro study using colorectal adenocarcinoma Caco-2 cells, the investigators found that oxidative DNA lesions could be triggered by enterohemorrhagic *E. coli* (EHEC or STEC) [54]. Of note, *E. coli* strains such as enterohemorrhagic *E. coli* and enteropathogenic *E. coli* can cause the formation of attaching and effacing (A/E) intestinal lesions, which is dependent on the bacterial type III secretion system (T3SS), and the development of DNA lesions. In a recent report, a significantly higher prevalence of enteropathogenic *E. coli* was detected among colon cancer patients compared to healthy participants [55]. Intriguingly, it is believed that *E. coli* might be involved in impairing the gut–vascular barrier at the site of neoplastic lesions, which could create a favorable condition for disseminating cancer cells or distant metastasis [56]. It is worth noting that *E. coli* was one of the initial bacteria that were thought to be connected with colon cancer pathology [Table 2].

## 3. Challenges with *E. coli* Management

Regarding the global antimicrobial resistance problem, the World Health Organization has specifically listed certain drug-resistant bacteria as serious public health threats; one of them is *E. coli*. Obviously, the epidemiology of the human–animal antimicrobial resistance relationship is exceptionally intricate. In the environment, antibiotic residues and *E. coli*, along with other bacteria, are commonly spread primarily with manure from food-animal production industrial farms. Logically, these byproducts affect the environmental bacteria, including microorganisms in wild fauna, which can be a source (or reservoirs) of drug-resistant bacteria [67]. In a study conducted in Italy, the investigators collected ESBL-producing *E. coli* isolates from humans and food-producing animals [68]. They observed that CTX-M was the most common type in human and animal isolates. Nevertheless, they concluded that ESBL gene transfer is possible from animals to humans. Another study in Tanzania analyzed the samples of feces from households and adjacent livestock, as well as soil and water in urban and peri-urban areas [69]. In 52% of household–livestock clusters, ampicillin- and tetracycline-resistant *E. coli* isolates were detected. The transfer of fecal bacteria among humans, cattle, soil, and water near livestock farms might happen frequently. Similarly, a study in Ethiopia found genetically similar *E. coli* (O157, a Shiga toxin-producing strain) in cattle, beef, and humans [70].

A research group in India collected samples from poultry farms (fecal matter, litter, and neighboring agricultural soil) and patients with urinary tract infections [71]. Interestingly, *E. coli* isolates from patients and poultry environments showed a similar resistance pattern for antibiotics such as amikacin, amoxicillin, ampicillin, and ofloxacin. Another study in the Netherlands, which analyzed ESBL *E. coli* isolates from broilers and staff of broiler farms, observed the transmission of bacterial strains, along with horizontal plasmid and gene transfer among broilers, workers, and their family members [72]. On the other hand, a study in New Zealand revealed that transmission within the same household (persons and pets) might contribute to the spread of ESBL- or AmpC beta-lactamase (ACBL)-producing *E. coli* in the community [73]. Moreover, Marchetti et al. concluded that *E. coli* isolates from dogs in Argentina could be a potential source of antibiotic resistance [74].

For the features mentioned above, it is evident that *E. coli* has highly complex cellular mechanisms and a great capacity for adaptation. Consequently, antimicrobial stewardship programs (ASPs) in certain places showed mixed results [75,76]. Wang et al. evaluated bacterial resistance data from 350,699 patients during the 2011–2016 period, and they observed that the resistance rates of *E. coli* to fluoroquinolones (levofloxacin and ciprofloxacin) declined as a result of antimicrobial stewardship, while the resistance rates to carbapenems (imipenem and meropenem) increased [75]. However, in an ASP on ESBL-producing *E. coli*, conducted in 214 primary health centers in Spain during 2012–2017, the intervention revealed a significant decrease in ESBL-producing *E. coli* infections along with an improvement in the use of antibiotics [77]. Similarly, another ASP in Spain, in 104 cases, recorded a favorable clinical outcome in urinary tract infections caused by ESBL-producing *E. coli* [78]. Furthermore, a study in Israel that included 6001 patients showed that ASP positively affected the antibiotic resistance rates of *E. coli* [79].

*E. coli* is widely present in the environment and in all mammals. Therefore, the ‘One Health’ approach is required for an effective ASP. With regard to this connection, functional cooperation is needed between several experts and regulatory bodies, e.g., veterinarians, physicians, pharmacists, food safety professionals, farmers, and environment/wildlife experts, as well as the relevant legal authorities at the national and international levels.

## 4. Link between *Chlamydia* and Lung Cancer Risk

**Figure 3:**
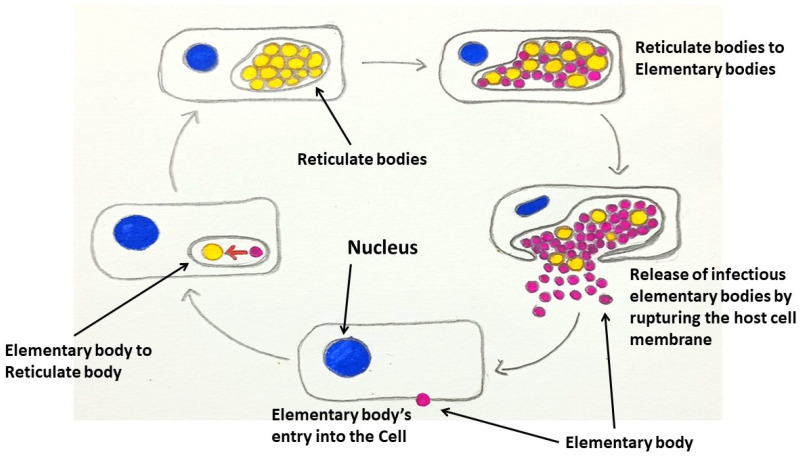
The life cycle of *Chlamydia*—development of the elementary and reticulate bodies.

*Chlamydiae* are Gram-negative obligate intracellular bacteria that maintain their life cycle in two phases: the infectious extracellular elementary body and the non-infectious intracellular reticulate body, which is a metabolically active replicative form (Figure 3). *Chlamydia* was initially thought of as a member of protozoa, subsequently a virus, and finally, it was discovered to be more analogous to *Rickettsia*, another Gram-negative obligate intracellular bacterium [80]. Although *Chlamydia* and *Rickettsia* are phylogenetically different, both share certain lifestyle characteristics, e.g., their intracellular survival, and have a wide range of hosts such as reptiles, birds, and mammals, which may result in zoonoses (Table 3). For this reason, many authors have tried to correlate these two groups of bacteria in different manners—for instance, genetic makeup, functions of a specific molecule, and utilization of cholesterol/lipids [81,82,83]. Nevertheless, *Chlamydiae* are unable to produce several essential biomolecules including ATP and components of nucleic acid and amino acid biosynthesis pathways; but a number of bacteria-associated molecules function as virulence factors, such as major outer membrane protein, polymorphic membrane proteins, lipopolysaccharide, and type III secretion systems [84]. In addition, *Chlamydia* infections can cause activation of various molecules/pathways, e.g., epidermal growth factor receptor, NF-κB pathway, IL-6 phosphatidylinositol 3-kinase, and mitogen-activated protein kinase, which are also connected with neoplastic pathological processes.

Early studies documented a connection between chronic *C. pneumoniae* infection and lung cancer [85,86,87]. In the cohort of Alpha-Tocopherol, Beta-Carotene Cancer Prevention Study, two serum samples were collected at a 3-year interval to detect *C. pneumoniae* infection before the diagnosis of lung cancer [85]. In this study, 230 male smokers with lung cancer, along with matched controls, were selected. The presence of chronic *C. pneumoniae* infection was detected in 52% of cases and 45% of controls. The incidence was specifically noticed in subjects younger than 60 years of age [85]. In the pathogenesis of lung cancer, smoking and chronic *C. pneumoniae* infection possibly act collaboratively [87,88,89,90]. On the other hand, a study on non-smoking women in China recorded that nearly 62% of patients with lung cancer (*n* = 192) and around 29% of healthy controls (*n* = 90) were immunoglobulin G (IgG) seropositive for *C. pneumoniae* [91]. In general, it has been observed that IgG and/or IgA seropositive titers against *C. pneumoniae* are a risk factor for lung cancer [92,93]. Interestingly, a case-control study in the USA on 593 lung cancer cases and 671 controls showed that elevated antibody titers for Chlamydial heat shock protein-60 (hsp60) were associated with an increased risk of lung cancer [94].

In a study in Austria, Aigelsreiter et al. examined the presence of *Chlamydiae* in five cases of lymphoma of mucosa-associated lymphoid tissue (MALT lymphomas) of the lung; all cases were positive for *Chlamydia psittaci* [95]. In another study in Greece, the investigators assessed surgically removed lung cancer tissue samples from 32 cases for the presence of *Chlamydia muridarum* and *C. trachomatis* [96]. In this study, 12.5% of cases were positive for *Chlamydia*. Of note, *C. muridarum* is a pathogen for mice. Interestingly, in a recent study on *C. muridarum* infection in knockout mice (*Il12rb2* KO and *STAT1* KO), a urothelial papilloma was developed in connection with this pathogen [97]. In another in vivo study, Wistar rats were divided into four groups; excepting the control group (*n* = 40) and carcinogenic benzo[*a*]pyrene group (BP, *n* = 46), the other two groups received repeated intratracheal administration of *C. pneumoniae* (only bacteria- *n* = 48, and with BP- *n* = 43) [98]. Incidences of lung cancer in the latter two groups were 14.6% and 44.2%, respectively, and 10.9% in the BP group. On the other hand, *C. pneumoniae* infection in pulmonary mesothelial cells (Mes1 cells) revealed induction of different cancer-linked genes, such as calretinin, Wilms tumor 1, and matrix metalloproteinase-2 [99]. Therefore, *C. pneumoniae* infection may favor the transformation of cells.

It is believed that the proliferation of *C. pneumoniae* in monocytes and macrophages in the lungs initiates pathogenesis by releasing elevated concentrations of different pro-inflammatory cytokines such as tumor necrosis factor-α (TNF-α), IL-1β, IL-8, as well as reactive oxygen species (ROS) [92,93]. For this reason, chronic *C. pneumoniae* infection and tobacco smoking could act synergistically to increase cancer risk. Nevertheless, pro-inflammatory cytokines and ROS can bring about chronic inflammation, which leads to cell injury and DNA damage. As a result, defects in the repairing process of cells might enhance the risk of mutation, which could also aggravate the risk of cancer [92,93]. In addition to the plausible role in tumorigenesis, *C. pneumoniae* perhaps affects the disease course. A study on patients with advanced non-small-cell lung cancer (stages III and IV) showed favorable results when treated with azithromycin in addition to chemotherapeutic agents, viz. paclitaxel and cisplatin [100]. Azithromycin is one of the commonly used antibiotics for the management of *Chlamydial* infections. A prospective hospital-based study evaluated 82 patients with primary lung cancer [101]. In this study, 75.6% of patients were positive for IgG antibodies against *C. pneumoniae*, 45.1% were positive for IgA, and seropositivity for both IgG and IgA was detected in 41.5% of cases. However, the study concluded that pre-treatment for *C. pneumoniae* infection may modify the health-related quality of life. On the other hand, a report demonstrated alterations in the levels of cytokines during treatment, depending on the status of *C. pneumoniae* infection [102]. For instance, there was a gradual increase in the concentration of transforming growth factor beta (TGF-β) during radiotherapy. Finally, after radiotherapy, TGF-β displayed a significantly higher concentration among *C. pneumoniae* IgG-positive patients with lung cancer, compared to the IgG-negative group.

## 5. Antimicrobial Issues with *Chlamydia*

*Chlamydial* infection is associated with the highest incidence of sexually transmitted bacterial disease worldwide. The World Health Organization estimated 128.5 million new infections with the *Chlamydial* pathogen in 2020 among adults (15–49 years). In addition, a high recurrence rate has been observed in cases of *Chlamydial* infection [103]. However, it is not clear whether this high rate of recurrence is caused by reinfection or persistent infection by antibiotic-resistant bacteria. In a report from China, higher rates of 23S rRNA gene mutations were found in the azithromycin treatment-failure group [104]. In addition, recent studies indicated antimicrobial resistance in *C. psittaci* and *C. trachomatis* (lymphogranuloma venereum) strains [105,106]. In contrast, several observations were not able to detect any treatment-resistant *Chlamydial* strains in clinical samples [107,108,109,110]. In a recent study from Thailand, a group of researchers identified antimicrobial resistance genes from *Chlamydia* in semiaquatic reptiles [111]. Specifically, there are a number of reports on the tetracycline-resistant *Chlamydia suis* in pigs and the possible horizontal transfer of resistance genes to other *Chlamydial* species [112,113,114]. In pig farms, tetracycline is used routinely, and this practice is responsible for the homotypic resistance to tetracycline (homotypic: where most organisms display resistance). Nevertheless, this type of resistance gene transmission was either undetected or inconclusive in wild boar populations [115,116].

In general, the determination of antibiotic resistance and the identification of relevant genes are performed in the laboratory/in vitro by serial passage of *Chlamydial* strains in sub-inhibitory (i.e., lower) concentrations of antibiotics. Nonetheless, the clinical implications of these in vitro findings are not clear [103]. Among patients, the variations in clinical outcomes could be due to other factors that are unrelated to antimicrobial resistance, such as hypoxia, interferon-gamma (INF-γ), IL-8, and macrophages [117,118,119].

Due to obligate intracellular parasitism, *Chlamydial* infections are generally diagnosed by certain complex methods such as cell culture, antigen-based detection techniques, or nucleic acid amplification tests (NAATs) [108]. However, there is no uniform methodology for antimicrobial susceptibility testing for *Chlamydiae*. Different laboratories use various cell lines, e.g., McCoy, HeLa (cervical cancer cells), and HEp-2, as well as BGMK and Vero (monkey kidney) cell lines. Although the McCoy cell line is commonly used, a number of these cells in many laboratories are mouse fibroblasts, not the original McCoy cells derived from human synovial tissue [120]. Similarly, the HEp-2 cell line which originally derived from laryngeal cancer cells was reported to be contaminated by HeLa cells [121]. It may be worth mentioning that there are cell line-dependent differences in in vitro antimicrobial susceptibility [122]. Nevertheless, the *Chlamydial* genes, which are linked with antimicrobial resistance in vitro, have been summarized in Table 4 [123,124].

## 6. Potential Prevention Aspect: Bacteriophages

Evidence suggests that *C. trachomatis* may increase the risk of cervical and ovarian cancers, in addition to causing other sexually transmitted diseases [125,126,127]. The situation has become more complicated due to the emergence of multidrug-resistant sexually transmitted infections all over the world. Along with *Neisseria gonorrhea*, growing antibiotic resistance problems have also been documented for other bacteria such as *Haemophilus ducreyi*, *Mycoplasma genitalium*, *Treponema pallidum*, and *C. trachomatis* [128]. Interestingly, both in vitro (using McCoy cells) and in vivo (in female BALB/c mice) studies showed an inhibitory effect of capsid protein Vp1 of chlamydiaphage φCPG1 on *C. trachomatis* serovar E strain [129]. Of note, φCPG1 is a lytic bacteriophage for *Chlamydia caviae*, which primarily causes inclusion conjunctivitis in guinea pigs. Currently, there are six known bacteriophages for different *Chlamydia* species, and they belong to the *Microviridae* family (chlamydiaphages Chp1–4, φCPG1, and ϕCPAR39—under the subfamily *Gokushovirinae*, and distantly related to *E. coli* bacteriophage ϕX174) [130]. These phages may have an extended host range; for example, ϕCPAR39 can infect *C. pneumoniae*, *C. caviae*, *C. abortus* (which causes miscarriages in ewes), and *C. pecorum* (which causes a wide variety of diseases in various animals) [130,131]. In one study, *C. pneumoniae* was grown in HeLa cells and infected with ϕCPAR39 [132]. The study recorded that ϕCPAR39 infection suppressed various protein syntheses of *C. pneumoniae*. In the same way, bacterial cell lysis was observed when *C. abortus* was cultured in BGMK cells and infected by chlamydiaphage Chp2 [133]. In another study, HeLa cells were used to grow *C. trachomatis*, which was subsequently infected with φCPG1 [134]. As expected, φCPG1 was able to inhibit the growth of *C. trachomatis* in a dose-dependent manner. These findings are fascinating, and perhaps the use of bacteriophages could be a potential method for future antimicrobial therapeutic strategies.

Since the initial discoveries around the early 1900s by Ernest H. Hankin (1865–1939), Frederick W. Twort (1877–1950), and Felix d’Herelle (1873–1949), scientists are now again thinking seriously about the issues of bacteriophages and methods to utilize them, either in combination with currently available antibiotics or alone, to manage the problem of multidrug-resistant bacterial infections. The first field trials of phage therapy were conducted in rural France against fowl typhoid, caused by Gram-negative *Salmonella gallinarum*, in 1919, as prophylactic measures [135]. Subsequently, phage therapy became popular during the 1930s, i.e., before the clinical use of penicillin among the masses. Although the discovery of antibiotics and their accessibility for people halted the interest in bacteriophage research and relevant therapeutic use, the study of bacteriophages and phage therapy was started in the Soviet Union when Felix d’Herelle moved to Tbilisi in 1934 and worked with his friend George Eliava (1892–1937) [135]. In the 1930s and 1940s, many research papers from the Soviet Union were dedicated to the topic of phage therapy in a wide variety of bacterial infections [136]. However, these studies were generally not accepted appropriately by the Western world. In recent times, particularly in the last decade, the situation has changed rapidly, due to the speedy emergence of multidrug-resistant bacteria across the globe, along with a decline in the process of new antibacterial discovery [137].

With regard to phage therapy, bacteriophages should have certain specific characteristics [138]. For example, only lytic bacteriophages can be considered in treating bacterial infections. Of note, in the case of a lytic (or virulent) group, new virions are released with the lysis of bacterial cells, whereas in the lysogenic (or temperate) group, viral genetic material is integrated with the host genome. On the other hand, unlike antibiotics, bacteriophages are able to kill only the specific bacteria that they recognize (i.e., without damaging commensal/symbiotic microorganisms). Lastly, administration of bacteriophages is easy, and only a few doses are needed, due to virus proliferation after the initial administration. However, it is necessary to resolve some important issues, e.g., proper identification of a useful bacteriophage for treatment, prevention of possible bacterial resistance against bacteriophages or phage-mediated antibiotic resistance (i.e., for lysogenic phages), and avoidance of an immune response against therapeutic bacteriophages [138,139]. Nevertheless, phage therapy could be an important component of ASPs in the near future.

Perhaps bacteriophages can efficiently kill bacteria in easily accessible zones, such as the body surface. In a recent study in a mouse model, lytic phage *Tequatrovirus YZ2* therapy has been shown to significantly enhance the healing of *E. coli*-infected skin wounds [140]. The study also noticed that the phage’s action was helpful in creating a favorable environment of cytokines, such as a decrease in IL-1β and TNF-α and an increase in the level of vascular endothelial growth factor. In an experiment during milk fermentation, coliphages DT1 and DT6, either individually or in combination, effectively reduced Shiga toxigenic *E. coli* without compromising lactic starter *Streptococcus thermophilus* [141]. On the other hand, the bactericidal effects of phages inside the body systems are not satisfactory thus far, possibly due to shortcomings in delivery techniques. An oral coliphage clinical trial using a T4 phage cocktail in acute bacterial diarrhea did not demonstrate any positive results, although there were no noticeable adverse effects [142]. The study included 11 T4-like phages, and 60% of the cases suffered from *E. coli* infections—the most common was enterotoxigenic *E. coli*. Of note, the group of T4 and related phages is considered a potential candidate in the treatment of infections with various *E. coli* strains [143]. Currently, seven enteric pathogenic *E. coli* have been described: enteroinvasive *E. coli*, enteropathogenic *E. coli*, enterohemorrhagic *E. coli*, enterotoxigenic *E. coli*, enteroaggregative *E. coli*, diffusely adherent *E. coli*, and adherent-invasive *E. coli* (AIEC) [144].

In the CEABAC10 transgenic mice whose intestine was colonized with the AIEC strain LF82, oral administration of the cocktail of three bacteriophages (LF82_P2, LF82_P6, and LF82_P8) significantly reduced the quantity of AIEC from the intestine [145]. Enterocytes of this transgenic mouse express CEACAM6 glycoprotein, and LF82 can bind with it. In another study, the investigators induced colitis with the AIEC strain LF82 in BALB/cYJ mice [146]. The investigators collected AIEC strains from clinical samples, as well as non-*E. coli* bacteria that are associated with a healthy microbiome. In this study, a cocktail of seven phages (LF82_P2, LF82_P8, ECML-119, ECML-123-2, ECML-359, ECML-363, and CLB_P2) was administered twice a day for 15 days, and the regimen prevented inflammation. It is worth mentioning that AIEC is frequently associated with inflammatory bowel disease.

## 7. Conclusions

Primitive gut-derived structures are the sites for a number of important cancers, and a few of them are thought to be connected with bacterial pathologies. For instance, among the derivatives of the primitive foregut, *Helicobacter pylori* is often associated with gastric pre-cancerous changes, and plays an etiological role in both adenocarcinomas and MALT lymphomas. In addition, except for a supposed relationship between chronic *C. pneumoniae* infection and lung cancer, *Salmonella typhi* may promote the risk of gallbladder cancer. In recent times, several lytic phages have been isolated against the abovementioned bacteria. The suitable therapeutic use of these bacteriophages, which may include appropriate genetic modifications, could be a promising strategy in preventive medicine/oncology. On the other hand, the situation in inflammatory bowel disease or colon cancer development is more intricate, due to the possible involvement of several bacterial species, e.g., *C. difficile*, *E. coli*, *Campylobacter* spp., *Chlamydia* spp., *B. fragilis*, and *Fusobacterium* spp. In this type of condition, perhaps the cocktail of various suitable bacteriophages could be evaluated for efficacy. Furthermore, as per the concept of the gut–lung axis, this kind of biological (nonantibiotic) therapeutic approach may confer the necessary requirements for a healthy lung. Of note, the gut–lung axis concept proposes a significantly influential role of intestinal microbiota communities and their alterations on pulmonary conditions, possibly through different immune cells and relevant cytokines. For the same reason, there is a need to develop prebiotics (such as inulin and pectin) and probiotics (such as *Lactobacillus* and *Bifidobacterium*) appropriately, i.e., other biological methods for disease prevention. Nevertheless, proper lifestyle changes such as healthy diets and avoiding tobacco use, along with the reduction in harmful bacterial growth by a nonantibiotic strategy, might be useful in lowering the incidences and management of a sizable number of cancers.

## Figures and Tables

**Table 1:** Results of different clinical trials that showed the plausible role of probiotics (mainly *Lactobacilli* and *Bifidobacterium*) in the modification of gut microbiota and overall disease course among patients with inflammatory bowel disease.

Investigators	Study Design	Findings
Bengtsson et al., 2016 (Sweden) [26]	Patients with poor pouch function after restorative operative procedure for ulcerative colitis: probiotic group (*n* = 17, *Lactobacillus plantarum* and *Bifidobacterium infantis*) and placebo (*n* = 16).	There was no statistically significant difference between the two groups—probiotics did not improve pouch-associated dysfunction.
Fan et al., 2019 (China) [27]	40 patients with IBD: control group (*n* = 19, treatment with mesalazine) and probiotic group (*n* = 21, mesalazine + probiotics).	After treatment, fecal bacterial counts decreased significantly in both groups, but the number of *Lactobacilli* and *Bifidobacterium* increased significantly only in the probiotic group, which also showed lower levels of inflammatory markers (IL-6 and hs-CRP).
Fedorak et al., 2015 (Canada) [28]	Patients with Crohn’s disease within 1 month of ileocolonic resection and re-anastomosis: probiotic group (*n* = 59; received *Lactobacillus*—4 strains, *Bifidobacterium*—3 strains, and *Streptococcus salivarius*—*thermophilus*), and placebo (*n* = 60).	At day 90, there were no statistical differences between the probiotic and placebo groups. However, lower mucosal levels of inflammatory cytokines (e.g., IL-1β and IL-8) and a lower rate of recurrence in the probiotic group were noted.
Groeger et al., 2013 (Ireland) [29]	Probiotic feeding: ulcerative colitis (*n* = 22) for 6 weeks; psoriasis (*n* = 26), chronic fatigue syndrome (*n* = 48), healthy subjects with probiotic intake (*n* = 10), and healthy subjects with placebo (*n* = 12) for 8 weeks.	Probiotic consumption (*Bifidobacterium infantis*) resulted in diminished blood CRP levels in all disorders compared to placebo. Blood levels of IL-6 were decreased in ulcerative colitis.
Matsuoka et al., 2018 (Japan) [30]	195 patients with ulcerative colitis: placebo (*n* = 97) and probiotic group (*n* = 98, *Bifidobacterium breve* and *Lactobacillus acidophilus*).	There were no significant differences between the two groups. However, regardless of treatment, there was a significant reduction in *Bifidobacterium* species before relapse.
Palumbo et al., 2016 (Italy) [31]	Ulcerative colitis: 30 patients—mesalazine treatment, 30 patients—mesalazine + probiotics (*Lactobacillus acidophilus*, *Lactobacillus salivarius*, and *Bifidobacterium bifidus*). The treatment was continued for 2 years.	Patients with combination treatment displayed better improvement in comparison with the mesalazine group.
Shadnoush et al., 2015 (Iran) [32]	105 IBD patients with probiotic yogurt, 105 IBD patients with placebo, and 95 healthy persons with yogurt (intervention for 8 weeks).	The mean numbers of *Lactobacillus*, *Bifidobacterium*, and *Bacteroides* in the stool specimens among IBD patients receiving yogurt were significantly increased.
Tamaki et al., 2016 (Japan) [33]	Patients with active ulcerative colitis: probiotic group (*n* = 24, *Bifidobacterium longum*) and placebo (*n* = 23)—clinical trial for 8 weeks.	Probiotic supplementation decreased UCDAI scores.

IBD: Inflammatory bowel disease, Mesalazine (5-aminosalicylic acid): anti-inflammatory drug primarily used in ulcerative colitis, IL: Interleukin, hs-CRP: high-sensitivity–C-reactive protein, UCDAI: Ulcerative Colitis Disease Activity Index, which considers stool frequency, rectal bleeding, mucosal appearance, and clinical assessment (higher scores → severe disease).

**Table 2:** Selected recent studies that showed a link between *E. coli* infections and colon cancer development or associated clinicopathologic events.

Investigators, Place of Study, and Study Plan	Results in Brief
Butt et al., 2021 (6 Western European countries) [57] The European Prospective Investigation into Nutrition and Cancer (EPIC) study—pre-diagnostic serum samples from incident colon cancer cases and matched controls (*n* = 442 pairs).	Immunoglobulin A (IgA) seropositivity to *E. coli* protein Ag43 and IgG seropositivity to enterotoxigenic *Bacteroides fragilis* toxin BFT-1 were significantly associated with higher odds of developing cancer.
He et al., 2021 (China) [58] Fecal samples from 61 colon cancer patients and 72 normal persons were analyzed to evaluate the microbial diversity and composition.	In comparison to the normal control group, the numbers of *E. coli*, along with *Prevetella copri*, were significantly higher among cancer patients.
Iwasaki et al., 2022 (Japan) [59] 543 participants with colonic growth (22 cancer and 521 adenomas) and 425 participants with normal colon (controls). The study aimed to assess the prevalence of *E. coli* containing polyketide synthase (*pks*).	The percentage of *pks*+ *E coli* was 32.6% among cases (cancer and adenoma) and 30.8% among controls. There was no statistically significant association between *pks*+ *E coli* and colonic lesions.
Iyadorai et al., 2020 (Malaysia) [60] Fresh tissue samples from 48 colon cancer patients (both malignant and nearby non-malignant tissues) and 23 healthy controls (normal colon tissues) were collected for the detection of *pks*+ *E coli*.	8 colon cancer patients (16.7%) and 1 healthy control (4.3%) were found to be positive for *pks*+ *E. coli*.
Kamali Dolatabadi et al., 2022 (Iran) [61] Colorectal tissue samples were collected from 150 subjects during colonoscopy: 30 subjects with normal results, 30 subjects with normal results but a positive family history of colon cancer, 30 subjects with normal results but a history of colon cancer, 30 patients with adenocarcinoma-in-situ, and 30 patients with adenocarcinoma.	74 intracellular *E. coli* were isolated from all subjects (among them, there were 24 adherent-invasive *E. coli*/AIEC strains). The majority were isolated from rectal specimens (31/74). AIEC strains generally belonged to B2 and D phylogenetic groups.
López-Siles et al., 2022 (Spain) [62] AIEC phenotype was examined in 4233 *E. coli* isolated from the ileum and colon biopsy samples from 14 ulcerative colitis and 15 colon cancer patients.	Regarding the prevalence of AIEC, one cancer patient had AIEC-like isolates (6.7%), whereas 5 patients with ulcerative colitis harbored AIEC-like isolates (35.7%). All AIEC-like strains belonged to the B1 phylogroup except one, which was isolated from an ulcerative colitis patient.
Messaritakis et al., 2020 (Greece) [63] Microbial DNA fragments in peripheral blood were analyzed for the β-galactosidase gene of *E. coli* (along with the glutamine synthase gene of *B. fragilis* and DNA coding for 5.8S rRNA of *Candida albicans*) from 397 colon cancer patients and 32 healthy blood donors.	*E. coli* β-galactosidase gene was detected in 104 patients (26.2%). Detection of these microbial fragments was significantly associated with metastatic disease and prognosis.
Mirzarazi et al., 2022 (Iran) [64] Fecal samples were collected from 20 newly diagnosed colon cancer patients (before treatment) and 50 healthy persons.	55% of *E.coli* isolates from patients’ samples, and 26% of *E. coli* from healthy persons belonged to the B2 phylogenetic group. Moreover, the outer membrane protein A (OmpA) was overexpressed in the *E. coli* B2 phylogenetic group isolated from cancer patients, compared to the control group. The protein significantly decreased the expression of pro-apoptotic genes (Bax and Bak) and p53.
Rondepierre et al., 2024 (France) [65] Patients with colon cancer were evaluated for present and lifetime psychiatric problems. Out of 64 suitable patients, 12 participated. In this limited cohort, patients were followed up after surgery.	All patients with colonization by colibactin-producing *E. coli* presented with psychiatric disorders several years before cancer diagnosis.
Wachsmannova et al., 2018 (Slovakia) [66] Analysis was performed to identify the presence of intracellular bacteria in colorectal biopsy samples that were collected from 10 colon cancer patients, 10 cases with adenomas, and 9 healthy controls.	The noticeable increase in intracellular *E. coli* in patients with carcinoma and colorectal adenomas was statistically significant in comparison to biopsy tissue samples from controls.

**Table 3:** Two extensively studied Gram-negative obligate intracellular bacteria and associated diseases.

Bacteria	Species		Diseases
Chlamydiae	*Chlamydia trachomatis*	Trachoma biovar (serovars- A, B, Ba, C)	Trachoma: chronic conjunctivitis, visual impairment, blindness
		Genital tract biovar (serovars- D, E, F, G, H, I, J, K)	Non-specific urethritis, prostatitis, epididymitis, infertility, cervicitis, pelvic inflammatory disease, ectopic pregnancy, premature delivery, inclusion conjunctivitis, neonatal pneumonia; can promote HIV infection and cervical cancer pathogenesis
		LGV biovar (serovars- L1, L2, L3)	Lymphogranuloma venereum
	*Chlamydia pneumoniae*		Pharyngitis, sinusitis, ear infection, laryngitis, bronchitis, pneumonia; may contribute to asthma, arthritis, atherosclerosis, myocarditis and encephalitis
	*Chlamydia psittaci*		Respiratory infection (psittacosis), pneumonia; can initiate complications such as hepatitis, endocarditis, and inflammation of the nerves/brain
		Spread by (vectors)	
Rickettsiae	*Rickettsia rickettsii*	Ticks	Rocky Mountain spotted fever
	*Rickettsia akari*	Mouse mite	Rickettsialpox
	*Rickettsia conorii*	Ticks	Mediterranean spotted fever or Boutonneuse fever (ssp. *Conorii*, spread by dog tick); Indian tick typhus (ssp. *Indica*); Israeli spotted fever (ssp. *Israelensis*)
	*Rickettsia sibirica*	Ticks	North Asian or Siberian tick typhus
	*Rickettsia australis*	Ticks	Australian tick typhus or Queensland tick typhus
	*Rickettsia felis*	Flea	Pseudotyphus of California
	*Rickettsia japonica*	Ticks	Japanese spotted fever
	*Rickettsia africae*	Ticks	African tick bite fever
	*Rickettsia prowazekii*	Body lice	Epidemic typhus or sylvatic typhus (contact with flying squirrels)
	*Rickettsia typhi*	Fleas	Endemic typhus or murine typhus
	*Orientia tsutsugamushi* (family *Rickettsiaceae*)	Mites	Scrub typhus

HIV: human immunodeficiency virus; ssp.: subspecies. Musca flies can be a vector for trachoma (due to their capability to spread *Chlamydia trachomatis*). The trachoma biovar remains at the mucosal surface, whereas LGV infects the lymphatic system. Psittacosis is a zoonotic disease.

**Table 4:** *Chlamydial* genes that may negatively influence the effectiveness of antibiotic treatment.

Antimicrobial Agents	*Chlamydia* Species	Mutated Genes
Macrolides (azithromycin, erythromycin)	*C. trachomatis*	23S rRNA, *rplD*, *rplV*
	*C. psittaci*	23S rRNA
Tetracyclines (tetracycline, doxycycline, minocycline)	*C. trachomatis*	*tetA*, *tetR*, *rpoB*
Fluoroquinolone (ciprofloxacin, ofloxain, sparfloxacin)	*C. trachomatis*	*gyrA*, *parC*, *ygeD*
	*C. pneumoniae*	*gyrA*
Rifamycins (rifampin)	*C. trachomatis*, *C. pneumoniae*	*rpoB*
Aminoglycosides (gentamicin, streptomycin, kasugamycin)	*C. trachomatis*	*ksgA*
	*C. psittaci*	16S rRNA, *rpoB*
Lincomycin	*C. trachomatis*	23S rRNA
Fosfomycin	*C. trachomatis*	*murA*
Trimethoprim	*C. trachomatis*	*folA*

## Data Availability

No new data were created or analyzed in this study.
